# Intermittent fasting and metabolic dysfunction-associated steatotic liver disease: the potential role of the gut-liver axis

**DOI:** 10.1186/s13578-025-01406-w

**Published:** 2025-05-23

**Authors:** Zhaoxi Zhang, Alice Pik-Shan Kong, Vincent Wai-Sun Wong, Hannah Xiaoyan Hui

**Affiliations:** 1 School of Biomedical Sciences, The Chinese University of Hong Kong, Hong Kong SAR, China; 2 Department of Breast and Thyroid Surgery, The First Affiliated Hospital of Sun Yat-Sen University, Guangzhou, China; 3 Department of Medicine and Therapeutics, The Chinese University of Hong Kong, Hong Kong, China; 4 Hong Kong Institute of Diabetes and Obesity, Prince of Wales Hospital, The Chinese University of Hong Kong, Shatin, Hong Kong, China; 5 Li Ka Shing Institute of Health Sciences, Prince of Wales Hospital, The Chinese University of Hong Kong, Shatin, Hong Kong, China; 6 Institute of Digestive Disease and Department of Medicine and Therapeutics, State Key Laboratory of Digestive Disease, Li Ka Shing Institute of Health Sciences, Shenzhen Research Institute, The Chinese University of Hong Kong, Hong Kong SAR, China

## Abstract

Metabolic dysfunction-associated steatotic liver disease (MASLD) is a growing public health concern linked to the increasing prevalence of metabolic syndrome, including obesity and type 2 diabetes (T2D). MASLD remains a significant clinical challenge due to the absence of effective therapeutic interventions. Intermittent fasting (IF) has emerged as a promising non-pharmacological strategy for managing MASLD. Although the exact mechanisms underpinning the possible beneficial effects of IF on MASLD are not yet fully elucidated, the gut microbiota and its metabolic byproducts are increasingly recognized as potential mediators of these effects. The gut-liver axis may act as an important conduit through which IF exerts its beneficial influence on hepatic function. This review comprehensively examines the impact of various IF protocols on gut microbiota composition, investigating the resultant alterations in microbial diversity and metabolomic profiles, and their potential implications for liver health and the improvement of MASLD.

## Introduction

Metabolic dysfunction-associated fatty liver disease (MASLD), previously known as non-alcoholic fatty liver disease (NAFLD), is a significant public health issue that impacts around 30% of adults worldwide and is strongly linked to the increasing prevalence of metabolic syndrome including type 2 diabetes (T2D) and obesity [1]. MASLD is characterized by excessive lipid accumulation in hepatocytes, leading to metabolic stress and liver injury [2], and is frequently co-existing with overweight/obesity, insulin resistance, T2D, and metabolic dysregulation [3]. While most MASLD patients have isolated steatosis (metabolic dysfunction-associated steatotic liver [MASL]), its histopathological features have a gradual progression from simple steatosis to metabolic dysfunction-associated steatohepatitis (MASH) and to advanced liver fibrosis, cirrhosis and hepatocellular carcinoma (HCC), eventually leading to liver- or cardiovascular-related mortality [4]. Over the past two decades, MASLD has emerged as the most common chronic liver disease, with a global prevalence of 30.1% [5]. This rate continues to rise in tandem with the increasing prevalence of metabolic syndrome, T2D, cardiovascular disease, and other chronic metabolic conditions. By 2030, it is estimated that the number of MASLD cases could reach approximately 314.58 million, highlighting the significant impact MASLD is expected to have in the coming decades [6]. The exact pathogenesis of MASLD remains unclear, but it is believed to involve multiple parallel factors, including insulin resistance, hormones secreted by adipose tissue, nutritional factors, gut microbiota, as well as genetic and epigenetic influences [7]. These multiple converging pathogenic pathways pose challenges for drug development, and to date, resmetirom is the only pharmacological agent being approved by the United States Food and Drug Administration (FDA) since 14 March 2024, for the treatment of adults with MASH. Lifestyle interventions, aiming for weight loss with reduction of total caloric intake and alteration of the composition of macronutrients in the diet, remain to be the primary therapy for people with MASLD [8]. Emerging evidence highlights that the timing of food intake significantly influences the risk of MASLD, with factors such as skipping breakfast, irregular eating patterns, and nighttime eating playing key roles [9]. Conversely, changing dietary habits, including time-restricted feeding (TRF), can enhance overall quality of life and metabolic function, thereby reducing the risk of developing metabolic syndrome such as obesity [10]. Therefore, intermittent fasting (IF), which alternates between fasting and feeding states, may provide significant weight loss and benefits for liver and overall metabolic health in MASLD patients.

IF has various forms, such as alternate-day fasting (ADF), periodic fasting (PF), TRF, and fasting-mimicking diet (FMD), with the advantage of reducing caloric intake [11]. IF is simple, cost-effective, and has high participant adherence compared to other dietary interventions, making it a favored dietary approach for patients with metabolic syndrome [12]. A growing body of clinical evidence indicates that IF regimens, including the 5:2 fasting protocol and TRF, significantly reduce body weight, enhance glycemic control, and inhibit the progression of liver steatosis and fibrosis in MASLD patients (Table 1) [10, 13–15]. However, the specific influence of IF on MASLD warrants further exploration, as the gut-liver interaction is a crucial component. The gut-liver axis is the bidirectional communication between gut microbiota and the liver, where microbial metabolites influence liver function and metabolism, while the liver affects gut permeability, thus playing a critical role in metabolic regulation and liver disease progression [16]. As a crucial component of the gut-liver axis, the composition of the gut microbiota can be influenced by a variety of factors, including antibiotic usage, lifestyle choices, dietary habits, and host genetics. Both animal studies and human clinical trials have demonstrated that IF significantly modulates the gut microbiota, enhancing its overall health and fostering the enrichment of beneficial microbes linked to reduced inflammation and improved metabolic function (Table 2 and Table 3). Thus, the beneficial microbiota and their metabolites resulting from IF may promote metabolic processes and support liver health through interactions within the gut-liver axis.

**Table 1:** Impact of IF on Metabolic and Liver Status in MASLD Patients on RCT Studies

Type of IF	Study duration	Sample size	Body weight	Lipid profile	Glucose and insulin metabolism	Liver steatosis	Liver fibrosis	Other outcomes	Author, Year
ADF vs. Ctrl	8 weeks	43 (33:10)	↓	–	–	↓	↓	↓ALT	Johari et al. 2019 [15]
TRF vs. SC	12 weeks	32 (17:15)	↓		↓HOMA-IR	↓	↓	↓leptin	Hodge et al. 2014 [34]
5:2 IF vs. LCHF vs. SC	12 weeks	74 (25:25:24)	↓	↓LDL-C	↓HbA1c ↓HOMA-IR	↓	↓		Holmer et al. 2021 [35]
5:2 IF vs. SC	12 weeks	44 (21:23)	↓	↓Triglycerides	–	↓	↓	↓ALT ↓CRP	Varkaneh et al. 2022 [14]
5:2 IF vs. Liraglutide	24 weeks	61 (31:30)	↓	↓Triglycerides ↓LDL-C ↑HDL-C	↓FBG ↓HOMA-IR	↓		↓ALT	Xiao et al. 2022 [13]

no change, *ALT* alanine aminotransferase, *Ctrl* control, *CRP* C-reactive protein, *FBG* fasting blood glucose, *HbA1c* hemoglobin A1c, *HDL-C* high-density lipoprotein cholesterol, *HOMA-IR* homeostatic model assessment for insulin resistance, *IF* interment fasting, *LDL-C* low-density lipoprotein cholesterol, *ADF* alternate day fasting, *LCHF* low carbon high fat diet, *RCT* randomized controlled trial, *SC* standard care

**Table 2:** Preclinical studies investigating the effects of IF on the gut microbiome

Intervention	Animal Model	Biospecimen	Microbiological Analysis Method	Primary Results on microbiota	Author, Year
TRF with 7 h feeding per day for 28 weeks	Young (5 months) and aged (21 months) male Fisher 344 × Brown Norway hybrid F1 rats	Feces	*16 s rRNA*	Alpha diversity ↑ ↑: *Verrucomicrobia, RuminococcaceaeUCG-005, Ruminococcus_gauvreauil_group, Ruminococcus1, Akkermansia, Ileibacterium* ↓: *Actinobacteria, Patscibacteria, Romboutsia, Alloprevotella, Leuconostoc, LachnospiraceaeUCG-005, Lactobacillus, Turicibacter*	Hernandez et al. 2022 [47]
ADF for 7 months	Male B6.BKS(D)-Lepr^db^ /J homozygous Lepr ^db/db^ mice	Feces	16 s rRNA	↑: *Firmicutes, Lactobacillus, Oscillospira, Ruminococcus* ↓: *Bacteroidetes, Verrucomicrobia, Akkermansia, Bacteroides, Bifidobacterium*	Beli et al. 2018 [48]
ADF for 4 weeks	7-week-old female C57BL/6 J mice	Feces	16 s rRNA	Gut bacteria richness ↑ ↑: *Lactobacillaceae, Bacteroidaceae, Prevotellaceae*	Cignarella et al. 2018 [49]
Fast for 24 h	6-week-old male C57BL/6 mice	Feces	16 s rRNA	↑: *Akkermansia, Parabacteroides, Muribaculum, Eubacterium_coprostanoligenes, Muribaculaceae* ↓: *Lactobacillus, Bifidobacterium*	Zhang et al. 2023 [50]
IF consisting of 50% calorie intake for 4 days, followed by 4 days of fasting, and then another 4 days of 50% CR	C57BL/6 J mice	Feces	16S rRNA	↓ *Parabacteroides distasonis*	Li M et al. 2022 [46]
TRF with 8-h eating and 16-h fasting for 32 days	7-week-old Male C57BL/6 J mice	Feces	16S rRNA	α diversity ↑ ↑: *Firmicutes, Lactobacillales, Ligilactobacillus, Bacilli, Lachnospiraceae, Rikenellaceae, Alostipes* ↓: *Prevotellaceae*	Liu X et al. 2024 [51]
PF: 3-week feeding with AL and then 1-week feeding with 40% amount of AL	Ten-week-old female MMTV-TGF-a C57BL/6 mice	Feces	16S rRNA	In the IF group of adult mice: ↑: *Lachnoanaerobaculum, Peptococcus*	Keles NA et al. 2024 [52]
ADF for 10 weeks	Male C57BL/6 mice are fed a HFHC diet for 16 weeks to establish a MASH model	Cecum	16S rDNA gene sequencing	α-diversity ↑ ↑: *Lachnospiraceae, Peptococcaceae, Peptococcus, Butyricicoccus, Blautia* ↓: *Cyanobacteria, Lactobacillus*	Lin X et al. 2023 [21]
PF: a cycle of 11 days, with fasting on day 1, 3 and 5, and normal diet for the rest of the time for one month	12-week-old male C57BL/6 mice	Feces	16S rRNA	Gut microbiota richness ↑ ↓ *Parabacteroides distasonis*	Xie S et al. 2022 [53]
Low-protein low-carbohydrate FMD every other week for 8 weeks	Six-week-old male C57BL/ksJ-db (db/db) mice	Feces	16S rRNA	α diversity ↑ ↑: *Parabacteroides, Blautia* ↓: *Prevotellaceae, Alistipes, Ruminococcaceae*	Wei S et al. 2018 [54]
ADF for 3 weeks	male C57BL/6 mice	Feces	16S rRNA	α-diversity ↑ ↑: *Bacteroidetes, g_Alistipes* ↓: *Firmicutes, f_Bacillaceae, g_Bacteroides, g_Streptococcus, g_Corynebacterium*	Liu T et al. 2024 [55]
RAIF with a 16-h daily fasting for 30 days	6 week male BALB/c mice	Feces	16S rRNA	↑: *Lachnospireceae, Ruminococcaceae*	Su J et al. 2022 [56]
TRF with a 8-h daily feeding	DPP-IV-Fischer 344 male rats	Feces	16S rRNA	↑: *Lactobacillus spp., Akkermansia muciniphila*	Palomba A et al. 2021 [57]
5:2 fasting for 12 weeks	Adult Sprague–Dawley rats	Feces	16S rRNA	↑: *Acetatifactor, Peptococcus* ↓: *Prevotellaceae, Parabacteroides*	Luo Y et al. 2024 [58]
ADF for 10–12 weeks	3-month-old 5XFAD mice	Feces	16S rRNA gene sequencing	↑: *Firmicutes, Lactobacillaceae, Lactobacillus reuteri* ↓: *Bacteroidetes*	Pan RY et al. 2022 [59]
TRF with 18-h fasting daily, ADF and CR	Male C57BL/6 mice were fed a HFD for 6 week	Stool, ileal, and cecal contents	16S rRNA	α-diversity ↑ IF did not change the fungal populations, with the most abundant genera being *Candida, Penicillium, and Hanseniaspora. Actinobacteria* ↑: *Bifidobacterium, Lactococcus, Clostridiales, Mucispirillum, Desulfovibrio, Coprococcus in ADF; Ruminococcus, Christensenellaceae, Lactococcus* in TRF ↓: *Bilophila, Enterococcus* in ADF; *Bilophila* in TRF	van der Merwe M et al. 2020 [60]
TRF for 2 months	9-week-old, wild-type Kunming mice	Feces	16S rRNA	↑: *Firmicutes, Clostridia, Ruminococcaceae, Roseburia*	Hu D et al. 2019 [61]
12, 16 or 20 h fasting per day for 1 month	C57BL/6 J male mice	Feces	16S rRNA	16 h fasting: ↑*Akkermansia* and ↓*Alistipes*	Li L et al. 2020 [62]
TRF with 16 h daily fasting	8-week-old, wild-type male C57BL/6 mice	Ileum content and mucosa	16S amplicon sequencing	TRF restored diurnal dynamics of the ileal microbiome and transcriptome in HFD mice	Dantas Machado AC et al. 2022 [63]
24-h fasting	C57BL/6 mice	Feces	16S rRNA	↑: *Akkermansia, Bacteroidia, Deferribateres, Bacilli, Erysipelotrichia, Gamma-proteobacteria, Verrucomicrobiae* ↓: *Clostridia*	Graef FA et al. 2021 [64]
TRF with 15 h fasting daily for four weeks	Male Wistar rats	Feces	Bacteria culture	↑: *Bifidobacterium, Enterococcus*	Soares NL et al. 2021 [65]
Every other day feeding	Male Sprague Dawley rats	Feces	16S rRNA	↑: *Lactobacillus*	Prisco SZ et al. 2021 [66]
Every other day feeding	HFD obesity (6-week-old) C57BL/6 J mice	Feces	16S rRNA	↑: *Lactobacillus, Verrucomicrobiaceae, Akkermansia muciniphila*	Yang H et al. 2023 [67]
TRF with 18 h daily fasting 5 weeks	12-month-old male Wistar rat	cecum content	16S rRNA	↑: *Firmicutes, Bacteroidetes, Proteobacteria* ↓: *Actinobacteria*	Teker HT et al. 2023 [68]
Fasting for 24 h	6 to 8-week-old male C57BL/6 mice	Feces	16S rRNA	↑: *Ruminococcaceae-UCG-014, Akkermansia, Parabacteroides* ↓: *Helicobacter*	Huang W et al. 2022 [69]
ADF for 2 weeks or 20 weeks	C57BL/6 male	Feces	16S rRNA	Short-term IF (2 weeks): ↑*Bacteroides, Muibaculum and Akkermansia*; ↓ *Ruminiclostridium* Long-term IF (20 weeks): ↑ *Lactobacillus*; ↓ *Akkermansia*	Wu J et al. 2022 [70]
TRF with 16 h daily fasting	Six-week-old male C57BL/6 mice	Feces	16S rRNA	↑: *Lactobacillus, Mucispirillum, Acetatifactor, Lachnoclostridium*	Xia J et al. 2023 [19]
24 h fasting followed by 24 h feeding	female Balb/c mice (6–8 weeks old)	Cecum feces	16S rRNA	↑: *Alistipes, Rikenellaceae*	Ma RX et al. 2023 [71]
5:2 IF regimen	7-week-old C57BL/6 male mice	Feces	16S rRNA	↑: *Lactobacillus murinus OTU2*	Zhang Z et al. 2021 [72]
TRF with 16 h daily fasting for 8 weeks	Eight-week-old male Kunming mice	Feces	16S rRNA	↑: *Bacteroidetes, Lachnospiraceae, Ruminococcaceae* ↓: *Firmicutes*	Ye Y et al. 2020 [20]
TRF with 16 h daily fasting	Eight-week-old male C57BL/6 J mice	Cecum Feces	16S rRNA	↑: *Oscillibacter, Ruminococcaceae* ↓: *Lactobacillus, Lactococcus*	Zarrinpar A et al. 2014 [38]
ADF for 28 days FMD for 3 cycles	C57BL/6 wild-type male mice	Feces	qRT-PCR	ADF: ↑ *Akkermansia, Lactobacillus*; ↓ *Deferribacteres* FMD: ↑ *Akkermansia*; ↓ *Deferribacteres*	Gregor A et al. 2022 [73]
ADF for 18 weeks	Male C57BL/6 J mice (5-week-old)	Feces	16S rRNA	↑: *Bacteroidetes* ↓: *Firmicutes, Firmicutes/Bacteroidetes ratio*	Wang S et al. 2023 [74]
ADF for 8 weeks	Male C57BL/6 J mice	Feces	16S rRNA	α-diversity ↑ ↑: *Verrucomicrobia, Akkermansia muciniphila* ↓: *Firmicutes*	Lei S et al. 2024 [75]
5:2 IF regimen for 1 month	Drosophila melanogaster	Flies	16S qPCR quantification	↓: *Lactobacillus plantarum*	Catterson JH et al. 2018 [39]
ADF for 4 weeks	Healthy male Sprague–Dawley rats (2 months old)	Feces	16S rRNA	α diversity ↑ ↑: *Lactobacillus, Bacteroides, Alloprevotella, Prevotella 1, Rikenel-laceae RC9, Odoribacter, Catenibacterium spp.* ↓: *Prevotellaceae UCG-003, Lachnospiraceae NK4A136, Lachnospiraceae UCG-008, Prevotella 9, [Eubacterium] coprostanoligenes, Ruminococcaceae UCG-008, Ruminococcaceae UCG-003*	Wang J et al. 2023 [76]
ADF for 30 days	Male C57BL/6 J mice (6-week-old)	Entire intestinal contents	16S rDNA Gene	↑: *Firmicutes/Bacteroidetes ratio, Lactobacillus, Ruminococcus, Akkermansia* ↓: *Helicobacter, Prevotella, Parasutterella*	Liu J et al. 2021 [77]
ADFfor 28 days	Diabetic male BKS.Cg-Dock7^m^ + / + Lepr^db^ /J Homozygous Lepr^db/db^ mice	Feces	16S rRNA	α diversity ↑ ↑: *Lactobacillus, butyrate-producing Odoribacter* ↓: *Enterococcus, Streptococcus, and unknown Enterococcaceae*	Liu Z et al. 2020 [78]
ADFfor 30 days or 6 days	Six-week-old male C57BL/6N mice	Cecal contents	16S rRNA	↑: *Firmicutes Bacteroidetes ratio, Lactobacillus reuteri*	Li G et al. 2017 [79]
4-day cycles of FMD	DSS-induced C57BL/6 mice (8-weeks-old, female)	Feces	16S rRNA	↑: *Lactobacillaceae, Bifidobacteriaceae, Erysipelotrichaceae, Allobaculum* ↓: S24-7	Rangan P et al. 2019 [80]
ADF for 10 weeks	Male SHRSP Wistar-Kyoto rats	Cecal content	16S rRNA	↑: *Bacteroides, Lactobacillus, Lachnospiraceae, Oscilltbacter* ↓: *Proteobacteria, Parasutterella*	Shi H et al. 2021 [81]

*ADF* Alternate day fasting, *AL* ad libitum, *CR* caloric restriction, *DSS* chronic dextran sodium, *FMD* fasting mimicking diet, *IF* intermittent fasting, *HFD* high-fat diet, *HFHC* high-fat and high-cholesterol, *PF* periodic fasting, *RAIF* ramadan-associated IF, *SHRSP* spontaneously hypertensive stroke-prone, *TRF* Time-restricted feeding

**Table 3:** Clinical studies investigating the effects of IF on the gut microbiome

Fasting mode	Study design	Study population	Country	Primary results on microbiota	Author, Year
5:2 IF for 24 weeks	HELENA Trial: RCT	IF group: 49 overweight or obese adults Control group: 52 overweight or obese adults	Germany	↑: *Lactobacillales, Bacilli*	Sowah SA et al. 2022 [43]
IF1-P: one fasting day (total of 36 h) and six feeding days per week IF2-P: two fasting days (60 h total) and five feeding days per week	Secondary analysis of a larger, registered trial	Overweight/obesity adults IF1-P: n = 10 IF2-P: n = 10	USA	IF1-P: ↑ *Ruminococcaceae Incertae Sedis, Eubacterium fissicatena;* $*Sellimonas* IF2-P: ↑*Ruminococcaceae Incertae Sedis*, $*Eubacterium ventriosum*	Mohr AE et al 2022 [82]
TRF: feeding < 12 h one day for 12 weeks	Non RCT	TRF group: 25 obese adults Non-TRF group: 24 obese adults	Italy	↑: *Lachnospiraceae, Parasutterella, Romboutsia*	Ferrocino I et al. 2022 [83]
TRF: fasting 8 h one day for 25 days	Non RCT	TRF group: 56 healthy males Non-TRF group: 24 healthy males	China	Gut microbial richness ↑ ↑: *Prevotellaceae, Bacteroideaceae*	Zeb F et al. 2020 [44]
FMD for 12 months	Auxiliary studies of RCT	FMD: 34 overweight or obese adults Daily caloric restriction (DCR): 25 overweight or obese adults	USA	↑: *Akkermansia*	Stanislawski MA et al. 2021 [84]
TRF: fasting at least 16 h one day for 27 days	Observational study	14 healthy women and 31 men	Pakistan	α diversity ↑ ↑: *Lactobacillus, Bifidobacterium, Lactococcus* ↓: *Pseudomonas triavilis, Brevibacillus limnophilus, Bacillus spp, Dorea spp*	Khan MN et all. 2022 [45]
Two calorie-restricted vegan days (max 1200 kcal/ day), followed by 5-days with a daily nutritional energy intake of 300–350 kcal/day, then refeeding for 3 months	Part of a RCT	Male and female patients with metabolic symdrome	Germany	↓: *Faecalibacterium prausnitzii, Eubacterium rectale, Coprococcus comes, Escherichia coli*	Maifeld A et al. 2021 [85]
One-week low-calorie, plant-based fasting program, followed by a probiotic intervention for 6 weeks	Observational study	13 overweight aduls	Austria	↑: *Lactobacilli, Enterobacteria, Bifidobacteria, Akkermansia*	Remely M et al. 2015 [86]
RF at least 28 days	Observational study	30 healthy adults	Iran	↑: *Bacteroides, Firmicutes*	Mohammadzadeh A et al. 2021 [87]
Buchinger fasting for 10 days	Observational study	16 healthy males	Germany	↑: *Bacteroidetes, Proteobacteria* ↓: *Firmicutes, Lachnospiraceae, Ruminococcaceae*	Mesnage R et al. 2019 [88]
Buchinger fasting for 5 days	Non RCT	Fasting group: 20 adults Non-fasting group: 31 adults	Austria	α diversity ↑ ↑: *Cyanobacteria, Verrcoumicrobia, Proteobacteria, Christensenella, Bifidobacteriaceae* ↓: *Euryarchaeota, Firmicutes, Actinobacteria, Firmicutes/Bacteroidetes* ratio	Lilja S et al. 2021 [89]
RF (17 h of fasting per day during a 29-day period)	Observational study	9 healthy adults (7 females and 2 males)	Turkey	↑: *Akkermansia muciniphila, Bacteroides fragilis*	Özkul C et al. 2019 [90]
eTRF (early TRF, eating during a period of no more than 8 h between 06:00 and 15:00, and fasting for the rest of the day); mTRF (mid-day TRF, eating during a period of no more than 8 h between 11:00 and 20:00, and fasting for the rest of the day); control group (eating ad libitum) for 5 weeks	RCT	Healthy adults without obesity: eTRF: n = 30 mTRF: n = 30 control groups: n = 30	China	eTRF: α-diversity ↑ mTRF: ↑*Leuconostocaceae*; ↓*Escherichia/Shigella, Weissella*	Xie Z et al. 2022 [23]
RF: fasting 16 h one day for 30 days	Prospective clinical study Non-RCT	Young healthy male adult cohort: n = 30 Middle-aged healthy cohort: Fasting group: n = 27; control group: n = 10	China	α diversity ↑ ↑: *Lachnospiraceae, Ruminococcaceae*	Su J et al. 2021 [91]
TRF: fasting 16 h per day for 25 days	Non-RCT	30 healthy men (18–30 y of age): TRF group: n = 15; non-TRF group: n = 15	China	↑: *Bacteroidetes, Prevotlla_9, Faecalibacterium, Dialister*	Zeb F et al. 2020 [92]
RF for 1 months	Observational study	Chinese group: 16 healthy adults Pakistani group: 18 healthy adults	China/ Pakistan	Chinese group: ↑*Dorea, Klebsiella, Faecalibacterium* Pakistani group: ↑*Sutterella, Parabacteroides, Alistipes* ↓: *Coprococcus, Clostridium_XlV, Lachnospiracea*	Ali I et al. 2021 [93]
ADF for 15 days	RCT	Multiple sclerosis patients undergoing relapse IF group: n = 8 Control group: n = 8	USA	↑: *Faecalibacterium, Lachnospiracea incertae sedis, Blautia*	Cignarella F et al. 2018 [49]
5:2 IF for 8 weeks	RCT	IF group: 21 obese adults Control group: 18 obese adults	China	↑: *Proteobacteria, Rumonococcaceae, Roseburia, Clostridium, Spirochaetes*	Guo Y et al. 2021 [27]
RF for 4 weeks	Observational study	Healthy or overweight adults 15 Pakistanis and 5 Nigerians	Korea	↑: *Bacteroides, Lactobacillus, Sutterlla, Ruminococcaceae UCG-005, Agathobacter, Fusicatenibacter, Lachnoclostridium* ↓: *Coprococcus, Lachnospiraceae NK4A136*	Jo Y et al. 2023 [94]
RF: fast for an average of 14–15 h daily during the 29-day Ramadan month	Observational study	10 overweight or obese male	Turkey	α diversity ↑ ↑: *Bacteroidetes, Proteobacteria, Bacteroidia, Alphaproteobacteria, Erysipelotrichi, Bacteroidales, Erysipelotrichales, Actinomycetales, Erysipelotrichaceae, Prevotella* ↓: *Firmicutes/ Bacteroidetes* ratio, *Firmicutes, Clostridia, Clostridiales, Ruminococcaceae*	Selen H et al. 2024 [95]
TRF with 16 h of fasting for 1 months and then received rifaximin and continued with TRF for another month	RCT	Obese aadults: IF group: n = 11 CR group: n = 11 KD group: n = 11 AL group: n = 11	Mexico	IF: ↑α diversity, ↑*Akkermansia muciniphila*, ↓LPS-producing bacteria (gram-negative species such as *Bacteroides caccae* and *Bacteroides eggertii*)	Guevara-Cruz M et al. 2024 [96]
RF with 17 h fasting per day for 29 consecutive days	Observational study	12 healthy adults (7 women and 5 men)	Turkey	↑: *Proteobacteria, Escherichia, Shigella* ↓: *Firmicutes, Blautia, Coprococcus, Dorea, Faecalicatena, Fusicatenibacter, Lachnoclostridium, Mediterraneibacter*	Saglam D et al. 2023 [97]
5:2 IF for three weeks	Observational study	72 healthy adults ranging from regular to obese	China	↑: *Parabacteroides distasonis, Bacteroides thetaiotaomicron*	Hu X et al. 2023 [98]

*ADF* Alternate day fasting, *AL* ad libitum, *CR* caloric restriction, *FMD* fasting mimicking diet, *IF* intermittent fasting, *KD* ketogenic diet, *LPS* lipopolysaccharide, *PF* periodic fasting, *RCT* randomized controlled trial, *RF* ramadan fasting, *TRF* Time-restricted feeding

This review aims to examine the structural and functional alterations in the intestinal microbiota after IF treatment, evaluate the evidence related to the risk of MASLD, and elucidate the potential mechanisms by which IF modulates metabolic status and hepatic health via gut microbiota interactions.

**Fig. 1:**
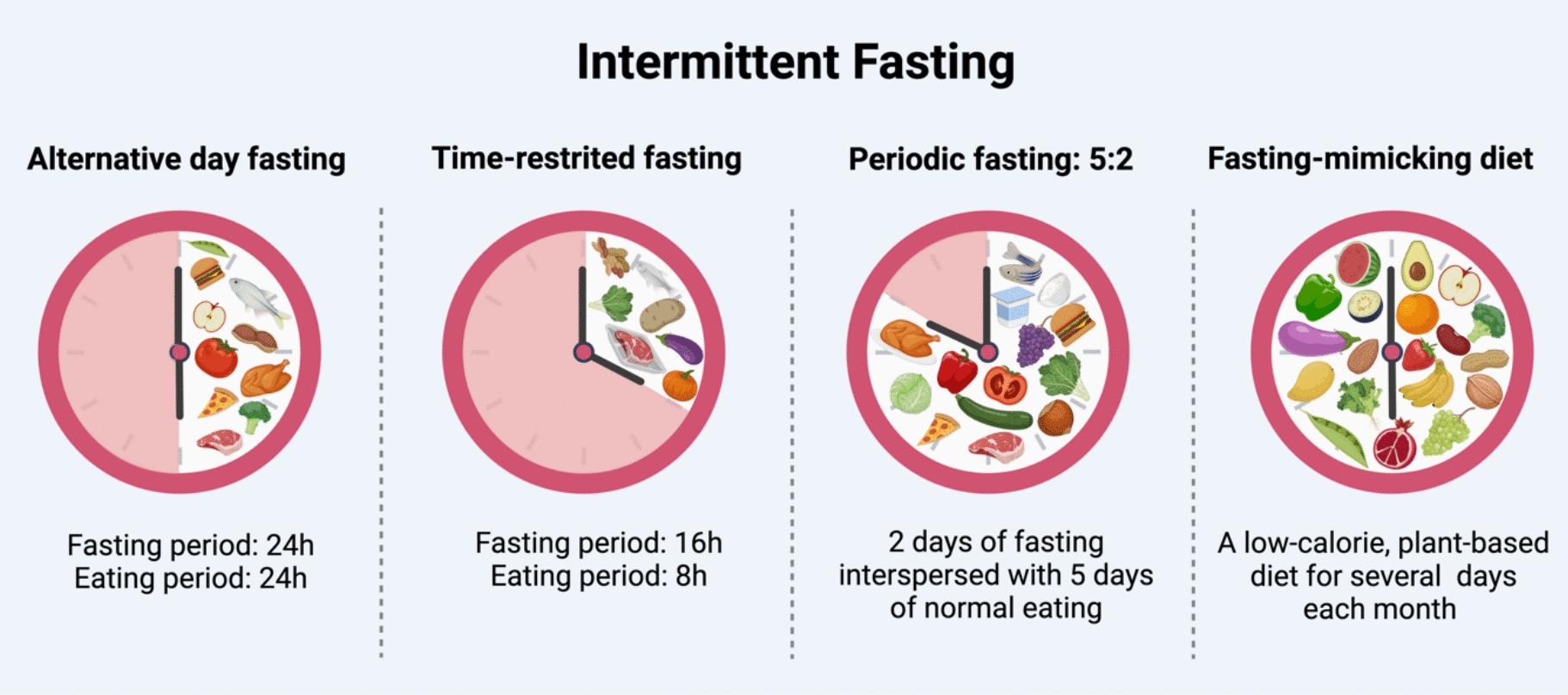
Types of IF. Four different types of IF were presented, including ADF, TRF, PF, and FMD

**IF**: IF is defined as an eating pattern that alternates between periods of fasting and eating, categorized into various types such as TRF, ADF, FMD and 5:2 diet (Fig. 1). While it has been associated with numerous health benefits including improved metabolic health, liver health, weight loss, enhanced cognitive function, and reduced risk of chronic diseases, potential risks may include nutrient deficiencies, disordered eating behaviors, and negative impacts on mood and energy levels [11, 12].

**TRF**: TRF, which limits daily food consumption to a window of 4 to 10 h, is considered a feasible fasting regimen for most individuals [17]. Ramadan fasting is a religious ritual for Muslims and is also considered a form of TRF. Over 1.5 billion Muslims fast or abstain from drinking from dawn (Sahur) to sunset (Iftar) during Ramadan, which lasts for 28 to 30 days [18], and therefore implementing IF and caloric restriction. Animal studies have demonstrated that TRF confers numerous benefits, including weight loss, obesity prevention, improved insulin sensitivity, reduced liver fat content, and prevention of hepatic steatosis and hyperlipidemia [19–21]. TRF has also been shown to reduce weight, increase insulin sensitivity, and lower blood pressure, providing benefits for patients with MASLD and non-alcoholic steatohepatitis [9]. Notably, previous human TRF studies have reported no serious adverse effects [22]. Research indicates that the efficacy of TRF is influenced by the timing of food intake, with early TRF (eTRF) yielding greater health benefits than mid-day TRF (mTRF) [23]. eTRF involves an 8 h eating window from 06:00 to 15:00, while mTRF restricts intake from 11:00 to 20:00. eTRF is associated with enhanced insulin sensitivity, lower fasting plasma glucose, reduced body mass and adiposity, diminished inflammation, improved liver health, and a more diverse gut microbiota compared to controls [23]. In contrast, mTRF does not demonstrate these benefits to the same extent.

**ADF**: ADF, also called every-other-day fasting (EODF), is defined as a strict 36 h period of caloric restriction (“fast day”), followed by a 12 h window of ad libitum food consumption (“feast day”) [24]. Several studies indicate that short-term ADF (less than 24 weeks) promotes weight loss and alters the risk of chronic diseases including MASLD [15, 24]. However, the long-term side effects of ADF (24 weeks or more), including hypoglycemia, gastrointestinal damage, and impaired bone metabolism, remain controversial in humans [25, 26].

**PF**: The 5:2 IF regimen is regarded as one of the least restrictive and the most popular forms of periodic fasting, involving two non-consecutive days of moderate to complete caloric restriction per week, while permitting ad libitum food consumption on the remaining five days [27]. Clinical trials indicated that 5:2 IF significantly reduced body fat, improved insulin resistance, and enhanced glycemic control, including glycated hemoglobin levels, in individuals with obesity and T2D [28, 29]. Gallage et al. found that the 5:2 regimen could prevent and improve MASH and fibrosis, while also restricting the progression of HCC in various diet-induced MASH and MASH-HCC models. PPARα and PCK1 are identified as hepatic mediators of the beneficial effects of fasting in MASH [30].

**FMD**: FMD is a low-calorie, plant-based diet that strictly limits animal protein intake and is cyclically implemented for several consecutive days each month. This periodic energy restriction mimics the metabolic patterns of prolonged fasting, yet it is easier to adhere to and considered safer than complete caloric restriction [31]. Human FMD consists of a 5-day regimen: on day 1, it provides 1,090 kcal (10% protein, 56% fat, and 34% carbohydrates), while days 2 to 5 offer a consistent formulation of 725 kcal (9% protein, 44% fat, and 47% carbohydrates) [31]. A 4-day FMD reduced the size of multiple organs and systems in C57BL/6 mice, and upon re-feeding, there was an increase in the number of progenitor and stem cells, along with enhanced regenerative capacity. In middle-aged mice, bi-monthly FMD cycles extend lifespan, reduce visceral fat, lower cancer incidence and skin lesions, rejuvenate immune system function, and slow bone mineral density loss. In aged mice, FMD cycles promote hippocampal neurogenesis, decrease IGF-1 levels and PKA activity, increase NeuroD1 expression, and enhance cognitive function. Furthermore, a pilot clinical trial showed that three FMD cycles reduced risk factors and biomarkers for aging, diabetes, cardiovascular disease, and cancer, without significant adverse effects, supporting the use of FMD to extend healthspan [31].

### The protective effect of IF on MASLD

IF appears to be a promising non-pharmacological intervention for the treatment of MASLD. While direct evidence supporting IF’s ability to delay MAFLD-to-MASH progression remains limited, animal studies demonstrate its ability to suppress critical pathological pathways—particularly inflammation, oxidative stress, and steatosis [19, 21]. These findings provide indirect mechanistic support for its potential to slow disease conversion. IF may reduce total cholesterol (TC) and triglycerides (TG) in the liver and improve the serum lipid profile by lowering TC, TG, and LDL-C serum levels [21]. Mice fed a high-fat high-cholesterol (HFHC) diet exhibited significantly elevated serum ALT and AST levels, alongside increased relative mRNA expression of inflammatory chemokine (MCP-1) and cytokine (TNF-α) in liver tissue. In contrast, IF significantly decreased serum ALT and AST levels, as well as the expression of hepatic inflammatory cytokine (TNF-α) [21], exerting the hepatoprotective effects and anti-inflammatory actions of IF. In a MASH mouse model, a ten-week IF intervention significantly reduced body weight, energy intake, and epididymal fat percentage, while alleviating hepatic steatosis, ballooning degeneration, lobular inflammation, NAFLD activity score, and insulin resistance [21]. Similarly, Li et al. found that fasting significantly improved obesity, insulin resistance, and hepatic steatosis [32]. A mimicked fasting diet could reduce MASLD and even slow the progression of glucose intolerance and liver injury to more severe stages [33]. Moreover, TRF intervention significantly alleviated obesity and MASH by restoring the rhythmicity of bacterial gena (such as *Lactobacillus, Mucispirillum, Acetatifactor, and Lachnoclostridium)* and intestinal amino acids [19]*.*

In human randomized controlled trials (RCTs), it has been confirmed that IF, particularly the 5:2 regimen, can improve liver cirrhosis and fibrosis in patients with MASLD (see Table 1). A clinical trial found that, among individuals diagnosed with MASLD, an 8-week ADF regimen significantly reduced hepatic steatosis, improved liver shear wave elastography, and lowered ALT levels compared to unrestricted eating [15]. Similarly, TRF has shown positive effects on liver transient elastography, visceral fat, and insulin resistance in MASLD patients [34]. In contrast to ADF and TRF, a greater number of high-quality RCTs indicate that the 5:2 diet significantly improves clinical symptoms in MASLD. Holmer et al. conducted an open-label RCT comparing the 5:2 diet with standard care (SC) in 74 MASLD patients, demonstrating that the 5:2 diet was superior in reducing steatosis, LDL levels, and body weight [35]. Additionally, a 12-week 5:2 intervention yielded significant improvements in liver stiffness (-1.8 kPa), ALT (-17.6 U/L), HOMA-IR, HbA1c, and LDL compared to baseline [14]. Another study involving clinically diagnosed MASLD patients showed that a 24-week 5:2 diet intervention resulted in marked improvements in body weight, lipid profiles, blood glucose levels, liver function parameters, and hepatic fat content assessed by controlled attenuation parameter (CAP) [13]. Based on current clinical evidence, the 5:2 diet may serve as the most promising therapeutic strategy for MASLD management.

### The effect of IF on gut microbiome

Dietary changes have rapid and lasting effects on the gut microbiota [36]. The structure and function of the gut microbiota exhibit circadian rhythm patterns, and disruptions in the host's circadian rhythm—such as irregular eating patterns—can impact bacterial populations, leading to dysbiosis [37]. Consequently, maintaining a stable gut microbiota is crucial, as IF promotes regularized eating and fasting patterns, thereby facilitating the restoration of a healthy circadian rhythm within the gut microbiome. In mouse models lacking circadian rhythms, IF has been shown to induce periodic activity within the gut microbiota [20, 38]. Catterson et al. found that the short-term IF “2:5 diet” during early adulthood significantly extended lifespan in Drosophila by alleviating late-life gut pathology and enhancing gut barrier function, suggesting that even brief periods of fasting can confer lifelong health benefits [39]. IF exerts a beneficial impact on the microbiome by enhancing microbial diversity and rhythmic activity, as well as modulating the abundance of specific bacterial taxa [40]. These effects are particularly advantageous in attenuating the adverse consequences of high-fat diets on the microbiome and reducing the prevalence of obesity-associated microbial communities [38]. Specifically, in experimental models subjected to high-fat dietary interventions, IF led to an increase in protective bacteria from the *Ruminococcaceae* family, while concurrently decreasing the abundance of pro-obesity taxa such as *Lactobacillus* [38]. However, Age, sex, and health status may influence the effects of IF on the gut microbiome. Discrepancies in the impact of IF on the gut microbiome may also arise between animal experiments and human clinical studies. Using large datasets to compare the gut microbiota of mice and humans, it was found that the two share similarities at the phylum level, but there were significant differences at the species level [41]. The gut microbiota of mice changed within a week due to dietary alterations, while changes in human gut microbiota occurred at a much slower rate [42]. Although there are differences between the gut microbiota of humans and mice, both mouse models and human studies have shown that IF can alter the gut microbiota, subsequently influencing metabolic processes and liver health [20, 21, 27, 43–46]. Still, the restructuring effects of IF on the gut microbiota may vary due to differences in animal strains, rearing conditions, baseline microbiome characteristics, and feeding durations, so we have summarized the effects of different IF protocols on the gut microbiome in animal experiments (Table 1) and human clinical studies (Table 2).

### The role of intestinal microflora in IF-induced improvement in MASLD

**Fig. 2:**
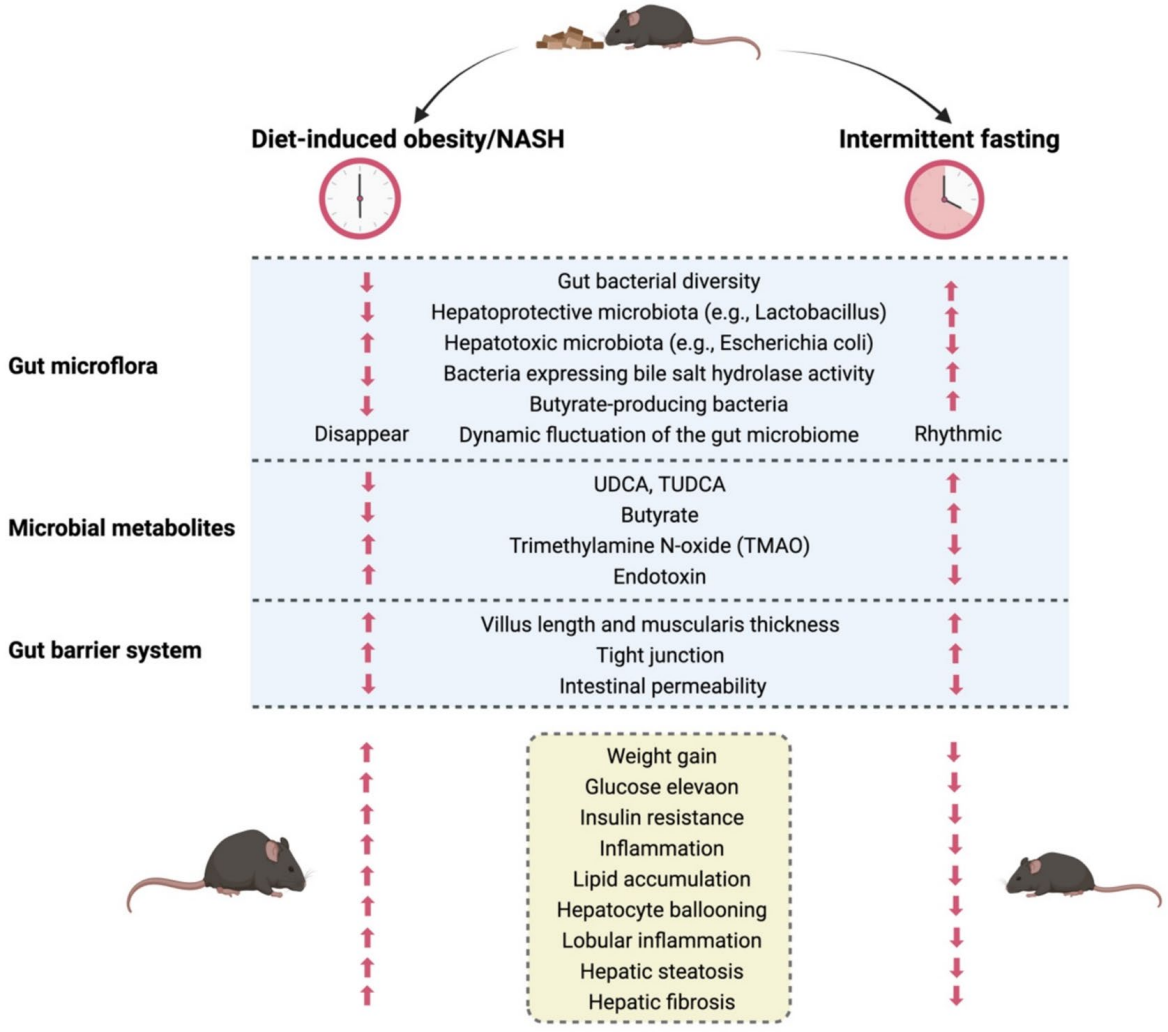
IF improves the gut environment, which in turn directly or indirectly protects against dysmetabolism and MASLD in diet-induced obesity/MASH mice. Mice subjected to a diet-induced obesity/MASH protocol were provided ad libitum access to a high-fat diet. These animals exhibited significant alterations in the gut microbiome, microbial metabolites, and intestinal barrier integrity, rendering them highly susceptible to dysmetabolism and MASH progression. IF restored cyclical fluctuations in gut microbiota composition, luminal metabolite profiles, gut signaling, and hepatic gene expression. Furthermore, fasting enhanced the abundance of beneficial gut bacteria and their metabolic byproducts while reinforcing intestinal barrier function. Collectively, these modifications conferred protection against dysmetabolism and MASH development

The gut microbiota plays a crucial role in energy metabolism and lipid homeostasis, as germ-free (GF) or microbiota-depleted mice show reduced sensitivity to diet-induced obesity and metabolic syndrome [99]. Gut microbiota potentially mediate the hepatoprotective and anti-inflammatory effects of IF according to substantial evidence from preclinical animal models (see Fig. 2).

### Gut-liver axis

Gut-liver axis denotes the reciprocal communication between the gut and liver, and it has been emphasized in recent clinical approaches. the intestine is tightly knit to the liver though hepatic portal vein [100], which circulates blood from intestine to liver. Under intricate regulation of gut barrier, essential substances in the enteric system pass through the intestinal lumen selectively, and finally empty into the bloodstream. However, inflammatory mediators, toxins, pathogens can enter the circulation if the gut barrier is compromised, whereby it loses the differential permeability and disturbs gut-liver homeostasis. Among these disturbances, the gut microbiota plays a predominant role [101]; its imbalance results in dysbiosis that drives liver disorders, and its metabolites and byproducts deteriorate hepatic processes.

Previous research showed that disorders of the gut, known as gut dysbiosis, can have a statistically significant relationship to certain pathophysiological alterations in the liver. Although the two organs are not physically in contact, their close proximity and several anatomical connections facilitate organ-to-organ crosstalk [102]. Disruption of gut microbiome homeostasis can lead to dysbiosis, characterized by a reduction in beneficial taxa, such as *Prevotella*, and an increase in pathogenic taxa, such as *Klebsiella pneumoniae* [103]. This imbalance can directly and indirectly impact the progression of MASLD [103]. Antibiotic-induced microbiota-depleted (AIMD) mice exhibited significant increases in glucose, triglycerides (TG), free fatty acids (FFA), and corticosterone, along with a decrease in insulin. These changes correlated with elevated liver transcripts of PEPCK and G6Pase, while the expression of Hes1, a negative regulator of hepatic TG production, was reduced [104]. Mechanistical studies have revealed that not only gut microbiota, but also cytokines, hormones and metabolites from the gut heavily influence common liver diseases. For instance, intestinal barrier impairment is regarded as a booster for developing MASLD [9], while aberrant gut microbial behavior is considered a hallmark for such condition. Also, the deficiency of microbiota leads to excessive corticosterone production in ileal intestinal epithelial cells (IECs), which in turn affects circadian rhythm components and systemic metabolism. GF mice exhibit increased TG and hypoinsulinemia [105]. Gut microbiota dysbiosis serves as both a pathogenic factor in MASLD onset and a regulator of its associated pathological processes. Transplanting gut bacteria from MASLD patients into mice can promote the development of liver inflammation and fibrosis associated with MASH by activating the accumulation of intrahepatic B cells through innate and adaptive immune mechanisms [106]. When the dynamic balance of gut microbiota is disrupted, the integrity of the intestinal barrier is compromised, resulting in bacterial overgrowth and translocation, which causes an influx of bacterial metabolites into the bloodstream that triggers inflammatory cascades and exacerbates metabolic dysregulation, thereby promoting the development of MASLD [16].

### Barrier systems against translocation of microorganisms

The gut barrier system is a complex network of physical, biochemical, and immune components that protect the intestinal lining from harmful substances and pathogens [16]. It includes a physical barrier of tightly joined epithelial cells, a mucosal layer, immune responses, and interactions with the gut microbiome, all crucial for maintaining gut health and homeostasis [16]. The integrity of the gut barrier is essential for maintaining gut permeability and preventing endotoxemia, and it is also linked to metabolic diseases and the subsequent development of MASLD [107, 108]. Brun et al. reported that obese ob/ob and db/db mice exhibited increased intestinal permeability, decreased expression of tight junction proteins occludin and ZO-1 in the intestinal mucosa, elevated circulating levels of pro-inflammatory cytokines, and the presence of portal endotoxemia [109]. Increased intestinal permeability and reduced tight junctions induced by high-fat feeding strongly led to elevated levels of endotoxemia, which contributed to impaired glucose intolerance, body weight gain, fat mass accumulation, elevated inflammation, and oxidative stress, further exacerbating metabolic disturbances [110]. Gut vascular barrier disruption is an early and common event in MASLD progression, promoting bacterial translocation to the liver [111]. Patients with MASLD showed significantly increased gut permeability due to disrupted intercellular tight junctions, as evidenced by reduced zona occludens-1 (ZO-1) expression in duodenal biopsy specimens, which may play a crucial role in the pathogenesis of hepatic fat deposition [107]. Children with MASLD demonstrated increased intestinal permeability, which correlates with the severity of hepatic involvement [108]. IF increased the villus length and muscularis thickness in diabetic mice, while also preventing intestinal leakage and reducing plasma LPS levels [78]. The expression of the tight junction protein claudin-1 in the intestinal barrier was elevated in the colon tissue of diabetic mice after IF treatment, which aligns with the improvement in intestinal permeability [78]. ZO-1 expression in the ileum was elevated in IF mice due to modulation by the gut microbiota [71]. IF also altered the composition of T cells in the gut lamina propria, resulting in a decrease in IL-17-producing T cells and an increase in regulatory T cells (Tregs) [49].

Plasma peptidoglycan: Peptidoglycan is a component of Gram-positive bacteria, with Firmicutes being a phylum of Gram-positive bacteria. Plasma peptidoglycan serves as a biomarker for gut barrier function. Alterations in the gut microbiota of IF mice are considered beneficial for gut health and the integrity of the intestinal barrier [48]. Diabetic mice exhibit elevated peptidoglycan levels, while IF diets reduce peptidoglycan levels during fasting [48]. This suggests that gut barrier integrity is compromised in diabetes, whereas the IF regimen (fasting days) can improve it. Jin et al. demonstrated that the administration of peptidoglycan alone is sufficient to induce liver steatosis and inflammation in mice on a normal chow diet, with NOD2 activation by peptidoglycan motifs driving the progression of these hepatic conditions [112]. Thus, the reduction of serum peptidoglycan induced by IF may represent a potential mechanism for alleviating MASLD.

### Gut bacterial diversity

Gut bacteria diversity refers to the variety and abundance of bacterial species in the gastrointestinal tract, which is essential for digestive health, immune function, metabolic balance, and the gut-liver axis [113]. IF significantly alleviated MASH and increased α-diversity in MASH mice induced by a high-fat and high-cholesterol diet [21]. Animals or Individuals who practice long-term IF have a fecal microbiota that is significantly more diverse than that of those who consume ad libitum diet [21, 23, 45, 47, 51, 54, 55, 60, 75, 76, 78, 89, 91, 95, 96]. An increase in gut microbiota diversity is regarded as a positive health indicator, as it enhances the ability to degrade and metabolize a variety of nutrients [45, 113]. A meta-analysis examining the association between gut microbiome composition and MASLD incorporated 54 studies with a total of 8,894 participants [113]. The findings revealed a significant reduction in α diversity indices among MASLD patients, specifically in Shannon and Chao 1 indices [113]. The majority of included studies in this review indicated a marked decrease in anti-inflammatory microbial taxa, such as *Ruminococcaceae*, alongside an elevation in pro-inflammatory taxa, including *Fusobacterium* and *Escherichia coli* [113]. Matthew et al. conducted an analysis of 847 fecal samples from 262 patients with acute or chronic liver disease using shotgun metagenomic sequencing and targeted metabolomics [114]. The study demonstrated that patients hospitalized due to liver disease exhibited reduced microbial diversity and a deficiency in bioactive metabolites, including SCFAs and bile acid derivatives, which can adversely affect immune defense and the integrity of the epithelial barrier [114].

### Hepatoprotective and hepatotoxic microbiome

Dysbiosis of the gut microbiome is associated with MASLD, typically characterized by a decrease in beneficial species (i.e., *Ruminococcaceae*) and an increase in pathogenic species (i.e., *Fusobacterium* and *Escherichia*) [113]. Another meta-analysis quantitatively evaluated the relationship between specific gut microbial taxa and the risk of MASLD, incorporating data from 15 studies involving fecal microbiome analysis of 577 MASLD patients and 688 healthy controls [115]. The results revealed that MASLD patients exhibited a significant increase in the abundance of *Escherichia coli, Prevotella,* and *Streptococcus*, while the levels of *Faecalibacterium, Coprococcus,* and *Ruminococcus* were markedly diminished compared to healthy controls [115]. Additional approaches targeting hepatoprotective bacteria, including fecal microbiota transplantation and the administration of probiotics, prebiotics, synbiotics, and engineered bacteria, may enhance metabolic function and reduce the risk of developing MASLD [16]. Based on Tables 1 and 2, both animal studies and human clinical trials demonstrate that various IF protocols promote the enrichment of anti-inflammatory, metabolic, and liver-beneficial microbiota, while concurrently reducing the abundance of pro-inflammatory bacterial populations.

### Hepatoprotective microbiome

IF can enrich the gut microbiota with beneficial bacteria for liver metabolism, such as *Lactobacillus, Bifidobacterium, Akkermansia muciniphila, Parabacteroides distasonis, Faecalibacterium prausnitzii,* and *Roseburia*, while reducing harmful gut bacteria associated with MASLD, such as *Escherichia coli*.

### Lactobacillus

*Lactobacillus* is frequently utilized as probiotics owing to their positive effects, which include the modulation of inflammatory immune responses and the enhancement of gut barrier function [116]. Both mouse models and human clinical studies have demonstrated that IF can lead to an enrichment of *Lactobacillus* in the gut microbiota [49]. The relationship between various *Lactobacillus* strains and obesity, as well as related metabolic disorders, has been extensively studied. Specifically, an increase in *Lactobacillus* levels may prevent obesity-related metabolic disturbances, potentially through alterations in bile acids within the gut lumen [117]. In one clinical study, MASLD patients administered *Lactobacillus acidophilus* three times daily for one month exhibited significant reductions in transaminases (AST and ALT), indicating that *lactobacilli* may help improve liver inflammation [118]. In another study, MASH patients who received *Lactobacillus rhamnosus* and inulin for 3 months experienced reductions in weight, waist circumference, and BMI, along with improved liver inflammation [119]. *Lactobacillus acidophilus* supplementation inhibited MASLD-HCC development in mouse models through the protective effects of valeric acid, which enhances intestinal barrier integrity and disrupts the Rho-GTPase pathway via GPR41/43 receptor binding [120].

### Bifidobacterium

*Bifidobacterium* is a safe and effective probiotic that can be used to treat liver diseases and can be effectively enriched after IF regimen [45, 80]. It successfully restores the balance of the gut microbiota and improves biochemical and clinical parameters in MASLD and cirrhosis [121]. The lack of *Bifidobacterium* is associated with an increased risk of metabolic-associated fatty liver disease in young patients with type 2 diabetes [122]. One species of the *Bifidobacterium* genus, *Bifidobacterium pseudolongum*, was the most significantly depleted bacterium in mice with MASLD-associated HCC [123]. This bacterium helped restore a healthy gut microbiome composition, improved gut barrier function, and provided protection against MASLD-HCC by secreting the anti-tumor metabolite acetate, which is transported to the liver through the portal vein [123]. *Bifidobacterium* metabolizes lactulose to produce high concentrations of acetate, contributing to the acidification of the intestinal lumen in MASLD patients and mice, which collectively help reduce the growth of antibiotic-resistant bacteria, such as vancomycin-resistant Enterococcus faecium [114].

### Akkermansia muciniphila

*Akkermansia muciniphila*, a member of the phylum *Verrucomicrobia*, is a mucin-degrading bacterium that resides in the mucus layer [124]. *A. muciniphila* may account for 3–5% of a healthy gut microbiome, and its relative abundance is negatively correlated with body weight [125]. It is an important gut symbiont for maintaining metabolic homeostasis and is currently recommended clinically as a novel probiotic for the treatment of obesity, diabetes, liver diseases, and other conditions [126]. *Akkermansia muciniphila* exhibits an inverse correlation with inflammation onset, adipose tissue metabolic alterations, and obesity-related metabolic disorders [127]. Oral supplementation of *Akkermansia muciniphila* can alleviate hyperinsulinemia and reduce plasma cholesterol levels, inflammatory biomarkers, and markers of liver dysfunction in overweight or obese adults with insulin resistance [128]. *A. muciniphila* is associated with the expression of genes involved in bile acid synthesis, metabolism, and transport, contributing to the maintenance of normal bile formation. The synbiotic combination of *A. muciniphila* and quercetin has been shown to improve early obesity and MASLD by reshaping gut microbiota and modulating bile acid metabolism [129]. Recent studies have found that treatment with *A. muciniphila* enhanced hepatic cholesterol absorption, BAs synthesis and transport, and improve the circulation and metabolism of BAs in the gut-liver axis [130] Moreover, A. muciniphila increased the conversion of CDCA to tauro-CDCA in hepatocytes, thereby inhibiting the expression of pro-inflammatory factors through TGR5, which helped reduce systemic inflammation [131]. In a murine model of liver cirrhosis, administration of *A. muciniphila* significantly attenuated hepatic fibrosis and hyperammonemia, and altered the bacterial composition in the small intestine [132]. In an auxiliary study of a RCT, 34 overweight or obese adults underwent a FMD for 12 months, and it was found that their gut microbiota showed a significant increase in *Akkermansia* [84]. A notable rise in the abundance of *A. muciniphila* was detected in nine healthy adults following a 29-day Islamic fasting, in comparison to baseline levels [90]. Additionally, other methods of IF, including TRF, have also resulted in the enrichment of the hepatoprotective bacterium *A. muciniphila* [86, 96].

### Parabacteroides distasonis

*Parabacteroides distasonis* is an anti-inflammatory gut bacterium that contributes to dietary fiber metabolism and is associated with gut health and various health benefits [133], which is also found significantly enriched after various IF rigemen [54, 98]. A three-week 5:2 IF program resulted in a significant enrichment of *P. distasonis* in the gut microbiota of 72 healthy adults [98]. In a preliminary analysis of data from 736 American Gut Project (AGP) samples, the abundance of Parabacteroides was significantly negatively correlated with body mass index (BMI) [134]. Among gut Parabacteroides, *P. distasonis* is defined as one of the 18 core members of the human gut microbiome and is believed to play an important physiological role in the host [135]; additionally, *P. distasonis* is relatively less abundant in individuals with obesity and non-alcoholic fatty liver disease [136]. According to previous reports, the enrichment of *P. distasonis* reduced weight gain, improve glycemic homeostasis, and correct obesity-related abnormalities, including hyperlipidemia and hepatic steatosis in ob/ob and high-fat diet (HFD)-fed mice through secondary bile acid-activated FXR signaling and succinate-activated intestinal gluconeogenesis [134]. *P. distasonis* is reduced in patients with hepatic fibrosis and may inhibits MASH through its metabolite pentadecanoic acid, while also improving liver fibrosis by modulating bile acid metabolism and regulating hepatocyte pyroptosis [137, 138]. Hu et al. found that a 3-week IF intervention might lead to an enrichment of gut bacteria rich in CAZymes, including *P. distasonis*, which helps alleviate obesity and its complications by producing succinate [98].

### Faecalibacterium prausnitzii

*Faecalibacterium prausnitzii* is a protective factor against MASLD in early-onset T2D, independent of age, sex, diabetes duration, and other confounding factors [122]. Clinical studies have shown that IF significantly increases the abundance of *F. prausnitzii* in the gut microbiota [86]. The relative abundance of *F. prausnitzii* is significantly reduced in MASH patients compared to healthy cohort [139]. Hu et al. administered 12 *F. prausnitzii* strains orally to HFD-fed C57BL/6 J mice for 12 weeks. Five strains—A2-165, LB8, ZF21, PL45, and LC49—significantly improved serum lipid profiles and mitigated glucose intolerance, adipose dysfunction, liver steatosis, inflammation, and oxidative stress in MASLD mice [140]. Additionally, the strains LC49 and LB8 notably enhanced the production of SCFAs and regulated the gut microbiota [140]. In MASH mouse models, supplementation with *F. prausnitzii* could improve glucose homeostasis, prevent hepatic lipid accumulation, inhibit liver injury and fibrosis, restore impaired gut barrier function, and alleviate liver steatosis and inflammation. Furthermore, *F. prausnitzii* treatment resulted in reduced hepatic lipid accumulation, decreased levels of AST and ALT, increased fatty acid oxidation in the liver, enhanced adiponectin signaling, improved insulin sensitivity in both subcutaneous and visceral adipose tissues, and a reduction in inflammatory responses in HFD mice [141].

### Roseburia

*Roseburia* is a genus of beneficial gut bacteria that ferments dietary fibers and produces SCFAs, promoting gut health and metabolic functions [142]. Observational studies and RCT have found that various IF protocols, including TRF, Ramadan fasting, and the 5:2 diet, lead to an increase in *Roseburia* levels in the gut microbiota of both healthy and obese individuals [27, 45, 143]. Patients with HCC showed a diminished presence of SCFA-producing bacteria, including *Roseburia* [144]. Furthermore, *Roseburia* is significantly reduced in individuals with obesity and T2D [145]. In a mouse model of alcoholic liver disease (ALD), administration of *Roseburia spp.* significantly mitigated hepatic steatosis and inflammation [146]. Notably, the flagellin protein of *R. intestinalis* activates Toll-like receptor 5 (TLR5), leading to increased expression of the tight junction protein occludin, which restores intestinal barrier integrity, and enhances IL-22 and REG3γ expression, promoting gut microbiota restoration [146].

### Enterobacteriaceae (Escherichia coli)

*Escherichia coli* is one kind of flagellated commensal bacteria residing in the human intestine, serving as an opportunistic pathogen commonly associated with complications in liver cirrhosis patients including ascites [147]. In a randomized-controlled bi-centric trial, a 3-month 5:2 IF regimen reduced *E. coli* levels in the gut of patients with metabolic syndrome, with a persistent depletion in *Enterobacteriaceae*, particularly *E. coli*, observed even at the end of a 3-month refeeding period [85]. In cirrhotic mice, *E. coli* translocated to the liver via the portal circulation by disrupting the integrity of the gut vascular barrier through farnesoid X receptor (FXR) signaling [148]. MASLD patients exhibited significantly higher serum immunoglobulin G titers against *E. coli* than healthy controls, indicating that *E. coli* may translocated to the bloodstream and subsequently actived systemic immune responses [149]. MASLD or MASH patients harbor significantly higher levels of *E. coli* in their gut microbiota and intestinal mucosa [150, 151]. Additionally, those with liver fibrosis exhibit even elevated *E. coli* levels compared to those without fibrosis [152]. *E. coli* activates TWIST1 via its flagellin-induced Toll-like receptor 5 (TLR5) and nuclear factor κB (NF-κB) pathways, facilitating the endothelial-to-mesenchymal transition of hepatic sinusoidal endothelial cells and promoting MASLD development [153]. The NF73-1 strain of Escherichia coli, isolated from the intestinal mucosa of MASH patients, translocated to the liver, activating the TLR2/NLRP3 pathway and leading to an increased population of M1 macrophages in the liver. These M1 macrophages, in turn, activated the mTOR-S6K1-SREBP-1/PPAR-α signaling pathway, thereby contributing to the progression of MASLD [151].

### Microbial metabolites and molecules involved in bidirectional communication between gut and liver

The IF-restructured gut microbiota also led to alterations in the microbial metabolites in plasma. Gut microbial metabolites, including BA, TMAO, and SCFA, are involved in the host's immune response and inflammation, and have a close association with liver health. IF may alter the circulating levels of microbial metabolites, which could help regulate the host's immune response and metabolic status.

### Bile acids

Primary bile acids (BAs) are synthesized from cholesterol in the liver, serving as emulsifiers for dietary fats and endocrine hormones that regulate glucose and lipid metabolism. Conjugated and unconjugated primary BAs are stored in the gallbladder and released into the small intestine, where they undergo structural modifications to form various secondary BAs, which demonstrate reduced host toxicity and diverse hormonal regulatory functions [154]. Microbial composition changes induced by IF can significantly modulate the enzymatic activity of these metabolic pathways including bile salt hydrolase and alter the bile acid pool composition [48]. IF treatment could significantly increase the enrichment of bacteria expressing bile salt hydrolase activity, such as Lactobacillus and Bifidobacterium [45, 80], which play crucial roles in deconjugating bile acids, modulating gut microbiota, influencing metabolic pathways, exhibiting antimicrobial properties, and contributing to overall host health [155, 156]. Certain Lactobacillus species produce bile acid hydrolase, which can conjugate bile acids that act as antagonists to the primary ileal bile acid receptor, FXR [117]. FXR-mediated enterohepatic bile acid signaling directly influences the activity of cytochrome P450 7A1 (Cyp7a1), also known as cholesterol 7α-hydroxylase, which is the primary enzyme responsible for de novo bile acid synthesis from cholesterol [157, 158]. Compared to HFD mice with ad libitum feeding, this enzyme exhibits higher activity levels in HFD mice after TRF, which correlates with the reduced serum cholesterol observed in TRF-HFD mice [159]. After IF treatment, there is an increase in primary, secondary, and tauroconjugated bile acids in the intestinal lumen of mice, indicating that IF enhances bile acid excretion [21]. In MASLD accompanied by cholestasis, there is a notable increase in hepatocellular inflammation, necrosis, and apoptosis, as well as fibrosis and disturbances in carbohydrate and lipid metabolism, all contributing to the progressive development of MASLD. IF treatment also significantly increases beneficial metabolites of bile acids, such as ursodeoxycholic acid (UDCA), in the serum, which may enhance metabolic health [21, 73, 75] UDCA is a hydrophilic bile acid commonly used to treat cholestatic liver diseases and has shown efficacy in MASH and MASLD [160, 161]. UDCA may alleviate liver inflammation in NASH mice by altering gut microbiota composition or enhancing bile acid signaling [162, 163]. Clinical studies indicate that UDCA can serve as a preventive measure against atherosclerosis, steatosis, and liver fibrosis in patients with non-alcoholic fatty liver disease [160]. IF protocol can also increase TUDCA [48, 78], which is formed from UDCA by combining with taurine in the liver [164]. TUDCA has been shown to act as an endogenous chemical chaperone, protecting liver cells from endoplasmic reticulum stress, thereby treating MASLD [165, 166]. Wang et al. found that TUDCA mitigated HFD-induced MASLD progression in mice by attenuating intestinal inflammation, enhancing gut barrier integrity, reducing intestinal fat transport, and modulating gut microbiota composition [164].

### SCFAs

Short-chain fatty acids (SCFAs), produced by the fermentation of indigestible fibers by gut bacteria, serve as the primary energy source for colonic cells and have been shown to enhance intestinal barrier function [167]. SCFAs include acetate, propionate, and butyrate. Butyrate serves as an energy source for colonic epithelial cells, while acetate and propionate act as substrates for lipogenesis and gluconeogenesis [168]. Butyrate is effective in preventing MASLD, MASH, inflammation, cancer, and liver damage, while also improving metabolic disorders such as insulin resistance and obesity [169]. IF treatment significantly improved butyrate-producing *Odoribacter* [78] and butyrate levels [87]. The main bacteria that produce SCFAs are from the *Ruminococcaceae* family [170], and some studies have shown that the abundance of *Ruminococcaceae* was significantly reduced in the feces of obese patients with MASLD and MASH [171–173]. However, both animal experiments and clinical studies related to IF have demonstrated that IF could increase the intestinal abundance of *Ruminococcaceae* (see Table 1 and Table 2). Supplementation with oral SCFAs or transplantation of SCFA-producing probiotics could enhance tight junction integrity in colonic cells [174]. Therefore, interventions aimed at increasing intestinal SCFAs levels may mitigate liver disease progression by reducing immune-related damage from portal transmission of bacterial endotoxins. A systematic review indicates that increasing intestinal SCFAs concentration may reduce gut permeability and liver injury, suggesting that upregulating intestinal SCFAs could be a promising therapeutic strategy for various liver disease models [175]. Reduced levels of SCFAs in the gut or bloodstream are also implicated in the progression of MASLD [176–178]. G protein-coupled receptor 43 (GPR43) is a receptor for SCFAs that regulates intestinal inflammatory responses. The SCFAs/GPR43 signaling pathway is believed to enhance gut permeability and minimize liver injury induced by microbial components and products [170]. Thus, the elevation of SCFAs concentrations induced by IF [78, 85, 87, 89] may constitute a pivotal mechanism contributing to the hepatoprotective effects associated with IF.

### TMAO

Trimethylamine N-oxide (TMAO) is one of the metabolites highly dependent on gut microbiota. Metabolize dietary precursors, such as L-carnitine, betaine and choline can be converted to trimethylamine (TMA) by gut microbiota in the intestine, which is subsequently transported to the liver via the portal circulation and metabolized to TMAO by flavin-containing monooxygenase 3 (FMO3) [179, 180]. TMA-producing bacteria, such as *Klebsiella pneumoniae* can produce TMA from choline via the CntC/D (choline-TMA lyase complex) and carnitine/butyrobetaine via the CntA/B (carnitine monooxygenase complex)—while *Proteus mirabilis* only possesses the CntC/D complex and can produce TMA solely from choline [181]. The increased abundance of TMA-producing bacteria may be one of the mechanisms leading to elevated plasma TMAO levels [182], and the adverse effects of TMAO on glucose and lipid metabolism may be mediated by the gut microbial metabolite TMA [183]. Additionally, it has been reported that healthy individuals with a high relative abundance of Firmicutes display increased circulating levels of TMAO [184]. The average TMAO level in a fasting state is 14.3 ng, compared to a baseline average of 27.1 ng in a feeding state [185].

IF can significantly lower circulating TMAO levels [186], potentially related to the duration of fasting: in overweight or obese adults, a one-day fast significantly reduces TMAO levels compared to a two-day continuous fast [82]. A dose–response meta-analysis indicates that for each 10 µmol/L increase in TMAO levels, the relative risk of all-cause mortality rises by 7.6% [187]. TMAO are significantly associated with the histological characteristics of MASLD and the risk of MASH in patients with T2D [188]. Prospective studies have shown that plasma TMAO concentrations are associated with all-cause mortality in MASLD patients [189]. Furthermore, a meta-analysis involving 34,000 patients revealed that the risk of fatal or non-fatal cardiovascular events in MASLD patients is 64% higher than in non- MASLD patients [190], potentially correlating with the severity of MASLD [191]. Additionally, among adults in hospitals and communities in China, levels of TMAO in the blood are associated with the severity of MASLD [192]. These findings suggest that TMAO may serve as an independent risk marker for MASLD. Indeed, TMAO promotes the development of MASLD by affecting bile acid metabolism, unfolded protein response and lipid metabolism. TMAO can reduce the size of the bile acid pool and inhibits the expression of key bile acid synthesis enzymes (Cyp7a1 and Cyp27a1) and bile acid transporters (Oatp1, Oatp4, Mrp2, and Ntcp) in the liver [180]. Furthermore, suppressing TMAO formation can upregulate hepatic bile acid synthesis enzyme CYP7A1 and alter the expression of liver genes crucial for bile acid feedback regulation, thereby preventing diet-induced hepatic cholesterol accumulation [193]. Another potential mechanism by which TMAO contributes to the development of MASLD may involve the unfolded protein response (UPR), a highly conserved pathway that monitors endoplasmic reticulum (ER) protein homeostasis, enabling cells to manage ER stress [194]. TMAO can bind to R-like endoplasmic reticulum kinase (PERK), selectively activating the PERK branch of the unfolded protein response, which induces the transcription factor FoxO1, a key driver of metabolic diseases in a PERK-dependent manner, and consequently leads to metabolic disturbances such as elevated blood glucose and insulin resistance in mice, both of which are significant risk factors for MASLD [195]. Additionally, studies have found that TMAO can promote lipid accumulation and fibrosis in vitro in hepatocytes by modulating the KRT17 gene [196].

### Microbiota-derived hepatotoxins

In individuals with liver disease, various gut microbiota and their cellular components frequently translocate into the portal circulation due to a disrupted intestinal barrier, subsequently reaching the liver [197, 198]. Certain compounds produced by gut bacteria can adversely affect liver function, resulting in inflammation and hepatocellular damage [198]. Among these, one of the most notable microbiota-derived hepatotoxins is lipopolysaccharides (LPS), which are the components of outer wall of Gram-negative bacteria and act as pathogen-associated molecular patterns (PAMPs) that activate Toll-like receptor 4 (TLR4), triggering pro-inflammatory signaling pathways [198]. LPS has been recognized as a putative trigger for the systemic inflammatory response [199]. Obese and diabetic mice exhibited increased intestinal permeability, decreased tight-junction integrity and elevated levels of LPS, which contributed to the development of insulin resistance [200]. Lipopolysaccharide-binding protein (LBP) is a liver-produced protein that binds bacterial endotoxin LPS, amplifies immune responses, and serves as a key biomarker for endotoxin activity. A prospective cohort study involving 920 adults found that LBP correlated with MASLD development and metabolic dysfunction in the general population [201]. Another study of 237 MASLD patients revealed that LBP was associated with NASH severity and liver fibrosis, with higher LBP linked to inflammation, fibrosis, and the TM6SF2 rs58542926 T allele—a genetic variant associated with impaired lipid metabolism and increased MASLD/NASH risk [202]. Children with MASLD also exhibited significantly higher serum endotoxin concentrations compared to the control group [203, 204]. Clinical study revealed that IF could significantly reduce the abundance of LPS-producing bacteria in obese human feces (Gram-negative species such as *Bacteroides caccae* and *Bacteroides eggertii*) and plasma LPS [27, 96]. This indicates that IF may be a potential therapeutic approach for improving endotoxemia in patients with MASLD, warranting further validation through future clinical trials.

### Dynamic fluctuation of the gut microbiome

The circadian clock in mammals is an endogenous system with a stable and accurate periodicity, systematically orchestrating the temporal oscillations of biological processes throughout a 24-h cycle to maintain physiological homeostasis [205]. Under the coordination of the host circadian clock and feeding behavior, the composition and function of the gut microbiota exhibit diurnal oscillations [206]. Firmicutes are most abundant during feeding in the dark/active phase, reaching their lowest levels during fasting in the light/inactive phase. In contrast, Bacteroidetes and Verrucomicrobia increase during fasting and decrease during feeding [63]. These periodic changes are associated with shifts in microbial community diversity, with alpha diversity rising during feeding and falling during fasting [63]. Diurnal oscillations in microbial composition drive rhythmic fluctuations in microbial metabolites, including SCFAs and BAs, which subsequently coordinate host metabolic homeostasis in a temporally specific manner [207]. These oscillations are essential for modulating peripheral circadian clocks and for the diurnal expression of metabolic regulators in the liver and gut that control glucose, cholesterol, and fatty acid homeostasis, as well as overall metabolic health [37, 208, 209]. Antibiotic-induced microbiota depletion abolishes the expression of gut circadian rhythm genes and disrupts intestinal epithelial cell (IEC) homeostasis [210]. Disruption of the gut microbiome's regular circadian rhythms can lead to dysregulation of the internal environment and metabolic disturbances, resulting in MASLD [19, 38, 207].

Diet-induced obesity (DIO) markedly elevates the risk of MASLD by facilitating hepatic fat accumulation and disrupting metabolic pathways, leading to insulin resistance, inflammation, and lipid metabolism alterations that culminate in liver damage and associated complications [211]. DIO can diminish or even eliminate the regular cyclical fluctuation of the gut microbiota community [19]. Conventionally raised mice lose their diurnal feeding patterns after ad libitum consumption of a HFD, with caloric intake during the light phase doubling compared to their pre-diet feeding behavior [212]. Disruption of this feeding pattern leads to dysregulated gut-liver axis circadian rhythms and dysregulation of metabolic regulators, resulting in increased adiposity, ectopic steatosis, and insulin resistance [19, 205].

Due to the peripheral circadian rhythm system's sensitivity to feeding patterns, dietary strategies such as IF are regarded as novel interventions for restoring oscillatory rhythms and alleviating metabolic syndrome. TRF is an emerging dietary intervention strategy that limits food consumption to a specific fixed time window of 8 to 12 h each day [213]. By consistently implementing diurnal feeding-fasting cycles, TRF robustly reinforce the rhythmicity of circadian rhythms and reprogram metabolic homeostasis and nutrient turnover to align with anticipated food availability, all without reducing total caloric intake or physical activity [214]. TRF prevents insulin resistance, reduces hepatic lipid accumulation, and maintains energy homeostasis in the progression of metabolic dysfunction, demonstrating efficacy even in core clock-deficient mice [214]. Additionally, TRF can partially restore rhythmic microbial oscillations in MetS, suggesting that it may improve metabolic rhythms and prevent further deterioration of MASLD through a microbiota-dependent mechanism [19, 20]. Mice subjected to a time-restricted high-fat diet exhibited less weight gain, milder liver steatosis, lower hepatic triglyceride levels, and distinct circadian rhythms in the hepatic expression of SIRT1, SREBP, and PPARα, along with altered circadian rhythms in the abundance of Bacteroidetes and Firmicutes, compared to those fed a normal diet ad libitum [20]. By restoring the rhythmicity of hepatoprotective genera, including Lactobacillus, Mucispirillum, Acetatifactor, and Lachnoclostridium, TRF mitigates HFD-induced weight gain, improves glucose tolerance, and reduces liver injury markers such as ALT, AST, alkaline phosphatase (ALP), and total cholesterol (TC) in MASH mice [19]. Furthermore, TRF decreases lipid droplet accumulation, fibrosis formation, extensive hepatocellular ballooning, severe steatosis, and inflammation, significantly lowering the NAFLD activity score in these mice [19]. This alleviation of MASH is contingent on the gut microbiota functioning at specific times of the day. Fecal microbiota transplantation (FMT) indicates that only the microbiota derived from the TRF feeding phase, not that from the TRF fasting phase, can protect mice from MASH and restore microbial rhythmicity [19].

The feeding/fasting cycle not only induces diurnal rhythmic oscillations in the gut microbiota but also affects the rhythmic fluctuations of its regulated metabolites, such as SCFAs and BAs. This, in turn, alleviates metabolic disorders and MASLD through rhythmic signaling within the gut-liver axis and the oscillatory expression of hepatic genes [215]. Dietary patterns such as IF can modulate the diurnal rhythms of SCFA-producing bacteria and SCFAs, resulting in significant alterations in metabolic states [216]. GPR43 is a short-chain fatty acid receptor that is abundantly expressed in white adipose tissue [217]. Upon activation by gut-derived SCFAs, it can regulate adipocyte sensitivity to insulin, thereby influencing lipid homeostasis [217]. Additionally, microbial-derived SCFAs can activate GPR41, impacting host energy balance by stimulating leptin production in adipocytes [218] and inducing enteroendocrine cell (EEC) secretion of glucagon-like peptide 1 (GLP-1) [219]. A study involving fully conventional mice with engineered native bacteria that express bile salt hydrolase (BSH)—an enzyme from the gut microbiome crucial for bile acid metabolism—demonstrates that BSH-overexpressing engineered native bacteria can induce alterations in circulating bile acids of obesity model mice, resulting in persistent improvements in glucose tolerance [220]. Regarding IF and bile acids, TRF partially restored the diurnal rhythms of the ileal microbiome and transcriptome, enhanced GLP-1 release, and modified the ileal bile acid pool and farnesoid X receptor (FXR)-FGF15 signaling pathway, thereby elucidating its metabolic and hepatoprotective benefits [63]. TRF can alter the HFD-induced ratios of unconjugated to conjugated bile acids between light and dark phases, primarily driven by variations in unconjugated bile acids [63]. SHP, encoded by Nr0b2 and a downstream target of FXR, is a key regulator in the bile acid signaling pathway [221] and is significantly overexpressed in TRF mice [63]. Additionally, circadian gene expression of bile acid transporters Fabp6 (IBABP), Slc51a (OST-α), and Slc51b (OST-β) was altered by HFD, but TRF could maintain gene expression levels for Fabp6 and preserved the circadian rhythmicity of Slc51a and Slc51b [63]. Ultimately, ileal bile acid signaling regulates de novo bile acid synthesis from cholesterol via CYP7A1, with hepatic expression of Cyp7a1 suppressed in HFD mice exhibiting elevated serum cholesterol levels, while TRF preserved normal Cyp7a1 expression and cholesterol homeostasis in these HFD mice [38, 63, 222].

### White adipose tissue browning

White adipose tissue (WAT) browning is a promising therapeutic strategy for treating obesity and related metabolic diseases, including MASLD [223, 224]. Browning WAT, called beige adipose tissue can facilitate negative energy balance and release endocrine signals known as batokines to prevent and reverse MASLD [224]. IF could promote browning of white adipose tissue by upregulating UCP-1 expression, improving insulin resistance in HFD-induced obesity [75]. Li and colleagues found that ADF selectively induced the browning of white adipose tissue, dramatically ameliorating obesity, insulin resistance, and hepatic steatosis in high-fat-diet-induced obese mice, with the gut microbiota playing a coordinating role in this effect [32]. Additionally, alternate-day fasting inhibited intestinal lipid absorption, promoted white adipose tissue browning, and reduced obesity and metabolic disorder risks via the microbiota-metabolite-fat signaling axis in HFD mice [67]. *Akkermansia muciniphila* reactivated browning of white adipose tissue, enhancing metabolic improvements to alleviate obesity [67].

**Fig. 3:**
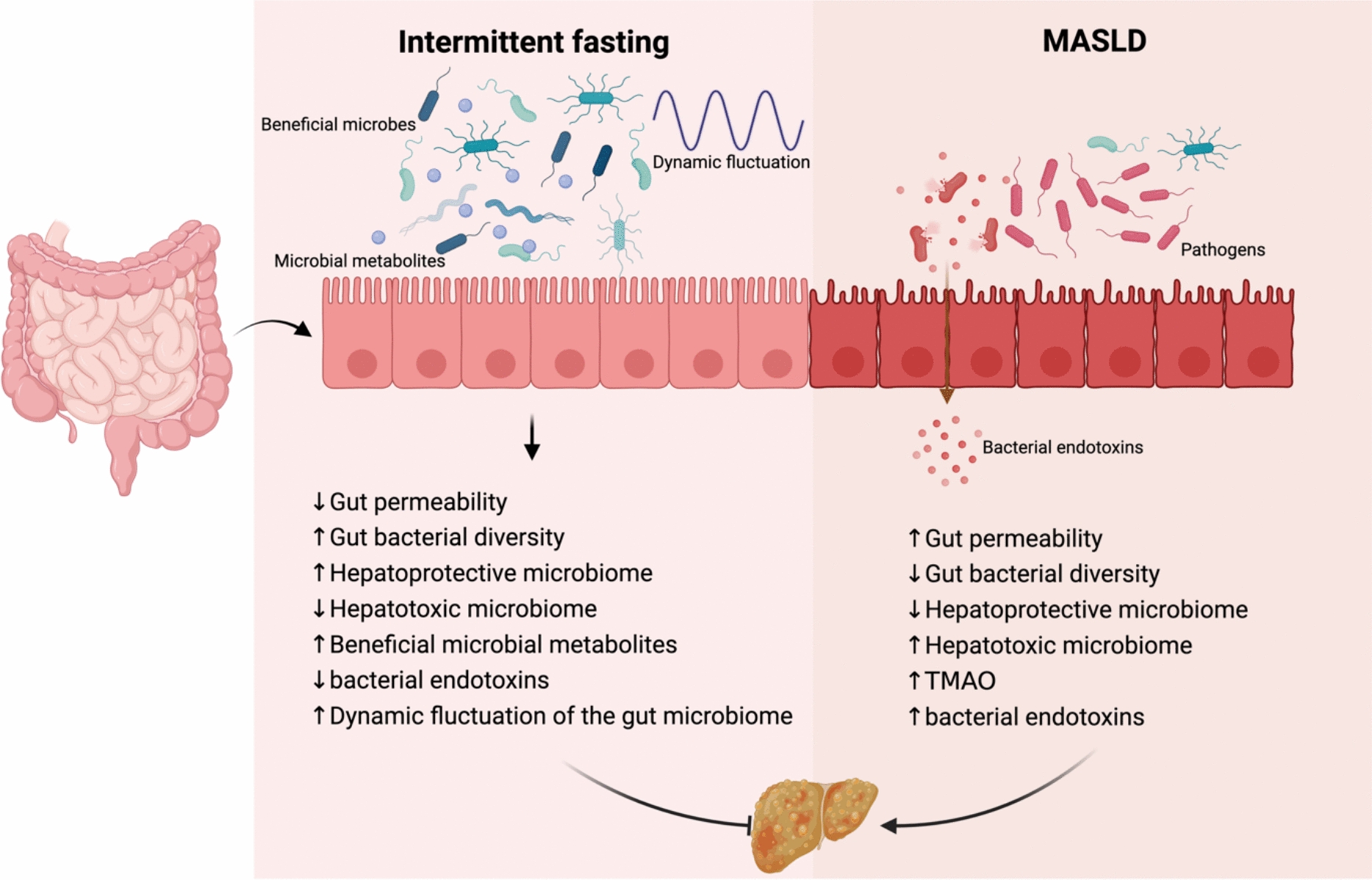
IF influences the intestinal environment and acts on MASLD. Patients with MASLD exhibit distinct gut microbiota dysbiosis, characterized by increased pathogenic bacteria (e.g., *Escherichia coli*), reduced α-diversity, depletion of beneficial species (e.g., *Lactobacillus* spp.), elevated hepatotoxic metabolites (e.g., TMAO), and impaired intestinal barrier function leading to endotoxemia. IF counteracts these pathological changes through multifaceted mechanisms: it restores microbial balance by enriching commensal taxa, enhances gut barrier integrity via tight junction protein upregulation, normalizes microbial metabolite profiles, and reestablishes circadian microbial rhythms. These coordinated modifications collectively attenuate MASLD progression by reducing hepatic inflammation, improving metabolic homeostasis, and decreasing endotoxin translocation, positioning IF as a promising gut microbiota-targeted therapeutic strategy for MASLD management

**Conclusion**IF emerges to be a compelling approach in the management of people with MASLD through facilitating beneficial alterations in the gut microbiota and promoting hepatic health. The intricate interactions within the gut-liver axis highlight its significance in mediating the therapeutic effects of IF (see Fig. 3). Future research should prioritize the identification of specific microbial taxa and their metabolites that contribute to the observed clinical benefits, alongside randomized controlled trials assessing the efficacy of different IF protocols on metabolic and liver health. By incorporating IF into clinical practice, we may enhance therapeutic strategies for MASLD, ultimately improving patient outcomes and addressing the growing burden of this condition.

### Challenges and future directions

Despite the promising potential of IF in MASLD management, several challenges remain. First, adherence to IF regimens can be highly variable among individuals, influenced by cultural, socioeconomic, and lifestyle factors, which may limit its widespread applicability. Second, the gut microbiota exhibits considerable inter-individual diversity, making it difficult to pinpoint universally beneficial microbial shifts or generalize findings across populations. Additionally, the precise mechanisms linking IF-induced microbial changes to hepatic improvements—such as whether effects are mediated by microbial metabolites, immune modulation, or barrier function—require deeper investigation. Long-term safety and efficacy data, particularly in vulnerable groups (e.g., patients with advanced liver fibrosis or diabetes), are also lacking. Addressing these gaps through rigorous clinical trials, personalized approaches, and mechanistic studies will be critical to harnessing IF’s full therapeutic potential.

## Data Availability

Data sharing is not applicable to this article as no new data were created or analyzed.

## Abbreviations

MASLD: Metabolic dysfunction-associated steatotic liver disease
MASH: Metabolic dysfunction-associated steatohepatitis
NAFLD: Non-alcoholic fatty liver disease
NASH: Non-alcoholic steatohepatitis
T2D: Type 2 diabetes
IF: Intermittent fasting
TRF: Time-restricted feeding
ADF: Alternate-day fasting
FMD: Fasting-mimicking diet
SCFAs: Short-chain fatty acids
TMAO: Trimethylamine N-oxide
FXR: Farnesoid X receptor
GPR: G protein-coupled receptor
LPS: Lipopolysaccharides
LBP: Lipopolysaccharide-binding protein
IgG: Immunoglobulin G
BMI: Body mass index
ALT: Alanine aminotransferase
AST: Aspartate aminotransferase
